# Expression of the receptor for hyaluronic acid mediated motility (RHAMM) is associated with poor prognosis and metastasis in non-small cell lung carcinoma

**DOI:** 10.18632/oncotarget.9554

**Published:** 2016-05-23

**Authors:** Dunrui Wang, Navneet Narula, Stephanie Azzopardi, Roger S. Smith, Abu Nasar, Nasser K. Altorki, Vivek Mittal, Romel Somwar, Brendon M. Stiles, Yi-Chieh Nancy Du

**Affiliations:** ^1^ Laboratory of Cellular Oncology, National Cancer Institute, National Institutes of Health, Bethesda, MD 20892, USA; ^2^ Department of Pathology and Laboratory Medicine, Weill Cornell Medicine, New York, NY 10065, USA; ^3^ Human Oncology and Pathogenesis Program, Memorial Sloan Kettering Cancer Center, New York, NY 10065, USA; ^4^ Department of Cardiothoracic Surgery, Weill Cornell Medicine, New York, NY 10065, USA

**Keywords:** lung cancer, receptor for hyaluronic acid-mediated motility, metastasis, prognosis

## Abstract

The receptor for hyaluronic acid-mediated motility (RHAMM) is upregulated in various cancers, but its role in primary and metastatic non-small cell lung carcinoma (NSCLC) remains to be determined. Here, we investigate the clinical relevance of RHAMM expression in NSCLC. RHAMM protein expression correlates with histological differentiation stages and extent of the primary tumor (T stages) in 156 patients with primary NSCLC. Importantly, while focal RHAMM staining pattern is present in 57% of primary NSCLC, intense RHAMM protein expression is present in 96% of metastatic NSCLC cases. In a publicly available database, The Cancer Genome Atlas (TCGA), RHAMM mRNA expression is 12- and 10-fold higher in lung adenocarcinoma and squamous lung carcinoma than in matched normal lung tissues, respectively. *RHAMM* mRNA expression correlates with stages of differentiation and inferior survival in more than 400 cases of lung adenocarcinoma in the Director's Challenge cohort. Of 4 *RHAMM* splice variants, *RHAMMv3* (also known as *RHAMM*^B^) is the dominant variant in NSCLC. Moreover, shRNA-mediated knockdown of *RHAMM* reduced the migratory ability of two lung adenocarcinoma cell lines, H1975 and H3255. Taken together, RHAMM, most likely RHAMMv3 (RHAMM^B^), can serve as a prognostic factor for lung adenocarcinomas and a potential therapeutic target in NSCLC to inhibit tumor migration.

## INTRODUCTION

Lung cancer is the leading cause of cancer-related deaths worldwide, and in the United States an estimated 221,200 new cases were expected to be diagnosed in 2015 [1]. Lung adenocarcinoma and squamous cell carcinoma, the two largest subtypes of lung cancer, account for 40% and 25–30% of non-small cell lung carcinoma (NSCLC), respectively [2]. Despite the improvements in diagnosis and therapy made during the past few years, the overall survival of lung cancer patients remains poor because the majority (57%) are diagnosed after the cancer has metastasized. The 5-year survival of patients with stage I NSCLC is 54.8% while the 5-year survival of patients with stage IV drops to 4.2% [3]. Some Food and Drug Administration (FDA) approved drugs for stage IV lung adenocarcinoma target molecular alterations including mutations in *epidermal growth factor receptor (EGFR)* and rearrangements of *anaplastic lymphoma kinase (ALK)* [4]. However, these molecular alterations are present in only 20% of patients with lung adenocarcinoma. Most patients are treated with chemotherapy regimens that are cytotoxic and ultimately fail. There is a critical need to develop predictive biomarkers of the metastatic potential of NSCLC, to prevent metastases from developing, and to treat metastatic lung cancer.

The receptor for hyaluronic acid-mediated motility (RHAMM), also referred to as HMMR/CD168/IHABP, was first identified as a 60–63 kD soluble binding partner of the extracellular matrix component, hyaluronic acid (HA), in the supernatant of chick fibroblast cultures [5]. In human tissues, low mRNA expression of *RHAMM* is found in lung and pancreatic tissue, and high mRNA expression of *RHAMM* is only detectable in testis, thymus, and placenta [6]. In contrast, RHAMM is upregulated in a number of cancers including pancreatic [7, 8], stomach [9], endometrial [10], bladder [11], colon [12], prostate [13, 14], breast [7, 15], head and neck [16], and glioblastoma [17]. RHAMM has been shown to have both extracellular and intracellular functions [18]. At the cell surface, RHAMM coordinates with other cell surface receptors to promote HA-induced cell growth and motility of multiple cell types [19–23]. Intracellular RHAMM, which has been found in human breast cancer cells [7], modulates cytoskeletal organization through interaction with microtubules and actin filaments [24] and contributes to activation of ERK [22, 25, 26].

The *RHAMM* gene encodes 18 exons and 4 variants are generated through alternative splicing [7, 24]. We previously demonstrated that RHAMMv3 (also known as RHAMM^B^) promotes metastasis of pancreatic tumors in mouse models [27]. In this study, we aimed to determine the expression and the prognostic value of *RHAMM* for primary NSCLC and metastatic tumors, and the expression levels of *RHAMM* variants in NSCLC.

## RESULTS

### Clinical and pathological features

A total of 156 patient specimens in tissue microarrays (TMA) were evaluated for the expression of RHAMM (Table 1). There were 91 (58%) females and the median age was 69 years old (interquartile range (IQR): 63 to 75 years). A majority of the patients (81%) were smokers. Of the 156 NSCLC cases evaluated, there were 127 adenocarcinomas, 22 squamous cell carcinomas, and 7 large cell or pleomorphic carcinomas.

**Table 1:** Demographic and clinical characteristics of RHAMM in primary non-small cell lung cancer

		RHAMM		
	Total *n* = 156	Negative staining *n* = 67	Positive staining *n* = 89	*p* value
**Age (Median), IQR**				
	69, IQR (63–75)	68, IQR (61–73)	70, IQR (64–77)	0.157
**Sex**				
Female	91	38 (42%)	53 (58%)	0.722
Male	65	29 (45%)	36 (55%)	
**Smoking**				
Yes	127	58 (46%)	69 (54%)	0.150
No	29	9 (31%)	20 (69%)	
**Histology**				
Adeno	127	59 (46%)	68 (54%)	0.180
Squamous	22	6 (27%)	16 (73%)	
Other (large cell, pleomorphic carcinoma)	7	2 (29%)	5 (71%)	
**Differentiation status**				
Well	32	20 (62.5%)	12 (37.5%)	0.030
Moderate	78	30 (38%)	48 (62%)	(significant)
Poor	36	12 (33%)	24 (67%)	
*Missing/unknown*	*10*	*5*	*5*	
**Pathological ‘T’ stages**				
T1	89	48 (54%)	41 (46%)	0.009
T2	53	15 (28%)	38 (72%)	(significant)
T3	3	0 (0%)	3 (100%)	
T4	11	4 (36%)	7 (64%)	
**Pathological ‘N’ status**				
Nx/No	130	59 (45%)	71 (55%)	0.169
N+	26	8 (31%)	18 (69%)	

IQR: interquartile range; Adeno: lung adenocarcinoma; Squamous: Squamous cell carcinoma; Nx, patients in whom the regional lymph nodes cannot be assessed; No: no regional metastases detected; N+: regional lymph node metastases.

### Correlation of RHAMM expression with demographic characteristics of patients with primary NSCLC

**Figure 1:**
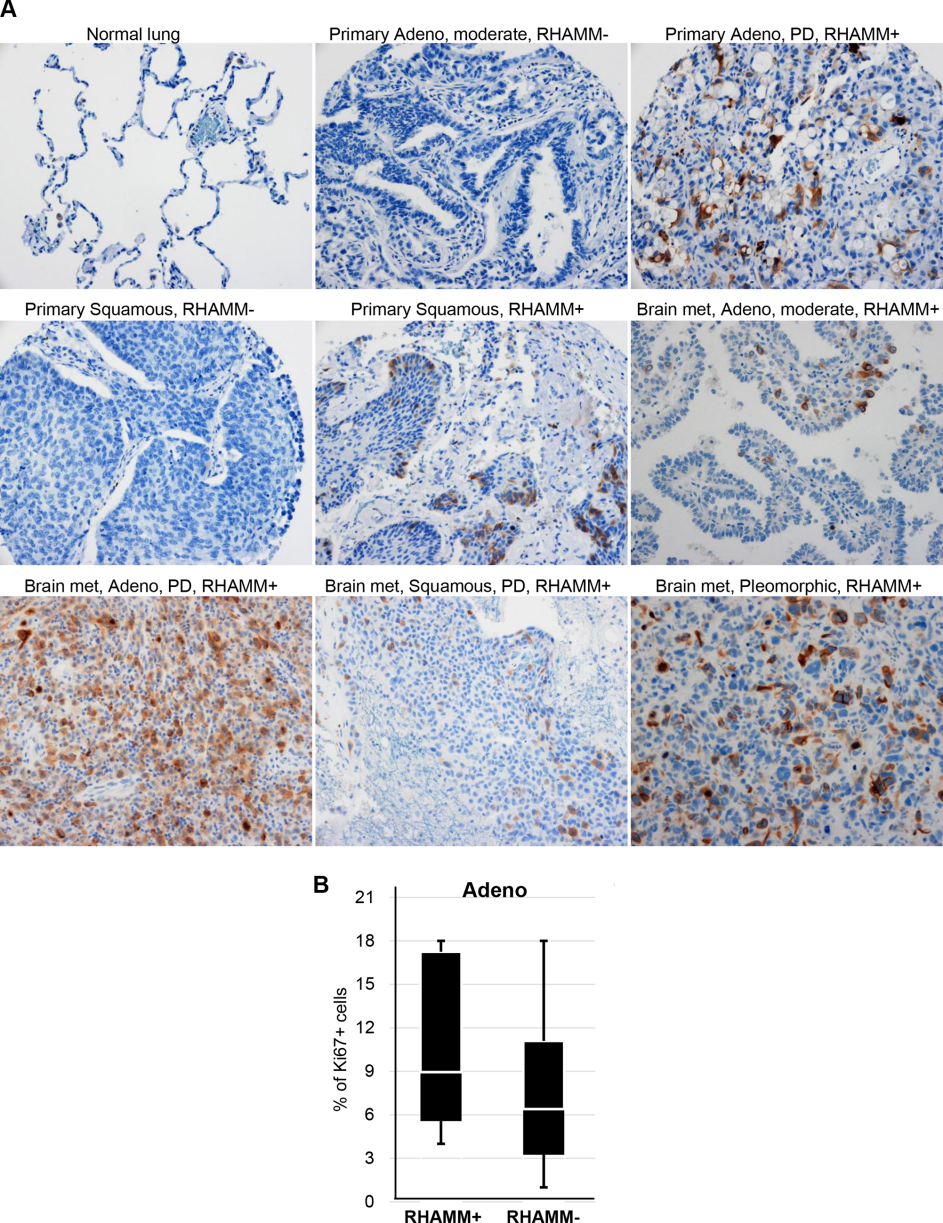
RHAMM protein expression is upregulated in NSCLC (**A**) Immunohistochemical staining of RHAMM in normal lung, primary NSCLC, and metastatic NSCLC. Adeno: lung adenocarcinoma; Squamous: Squamous cell carcinoma; moderate: moderately differentiated; PD: poorly differentiated; met: metastasis; Original magnification: 20×. (**B**) Proliferation rates measured by Ki67 immuohistochemistry did not significantly differ between RHAMM-positive and -negative primary lung adenocarcinomas.

Focal staining of RHAMM was found in 89 of 156 (57%) NSCLC (Figure 1). In contrast, normal lung (alveolar lining epithelial cells) and benign lymph nodes had no RHAMM staining (Figure 1 and data not shown). As shown in Table 1, there was no statistical correlation of RHAMM staining with age, sex, smoking history, or various histological types of NSCLC. Positive RHAMM staining was frequently found in poorly differentiated cancer (24/36, 67%) and in moderately differentiated cancer (48/78, 62%), but less in well-differentiated cancer (12/32, 38%). In addition, a high percentage of RHAMM-positive staining was found in more locally advanced tumors: T2 (38/53, 72%), T3 (3/3, 100%), and T4 (7/11, 64%) compared to T1 (41/89, 46%). There was an increasing trend of RHAMM expression in NSCLC with regional nodal spread compared to those that were node negative, although it did not reach statistical significance. (Table 1). Since currently available antibodies against RHAMM are not isoform-specific, isoforms cannot be distinguished by immunostaining. Furthermore, to study whether RHAMM expression correlates with proliferation of primary lung adenocarcinomas, we stained a subset of lung adenocarcinomas (*n* = 12) with a proliferation marker, Ki67. We found no significant correlation between RHAMM expression and proliferation as measured by Ki67 (*p* = 0.335, Figure 1B).

### RHAMM expression in metastatic NSCLC

We investigated the frequency of RHAMM positive staining in metastatic NSCLC from 23 patients (Table 2). The majority of metastases were adenocarcinomas (*n* = 17), but there were 2 squamous cell carcinomas and 4 NSCLC not otherwise specified. The most common metastatic site of these NSCLC was the brain followed by bone. RHAMM expression was intensely positive and occurred in 22 of 23 tissues (Figure 1) suggesting that RHAMM is significantly upregulated in metastatic NSCLC compared to primary NSCLC (Table 3).

**Table 2:** The majority of metastatic non-small cell lung cancers are RHAMM-positive

ID	RHAMM	Type	Metastatic site
922	+	Adenocarcinoma	Bone
927	+	Adenocarcinoma	Brain
928	+	NSCLC, NOS	Brain
929	−	Adenocarcinoma	Brain
930	+	NSCLC, NOS	Brain
931	+	NSCLC, NOS	Bone
932	+	Adenocarcinoma	Brain
934	+	NSCLC, NOS	Brain
935	+	Squamous cell carcinoma	Brain
939	+	Adenocarcinoma	Brain
940	+	Adenocarcinoma	Brain
947	+	Adenocarcinoma	Brain
949	+	Squamous cell carcinoma	Brain
951	+	Adenocarcinoma	Brain
954	+	Adenocarcinoma	Brain
956	+	Adenocarcinoma	Brain
963	+	Adenocarcinoma	Brain
964	+	Adenocarcinoma	Brain
965	+	Adenocarcinoma	Brain
966	+	Adenocarcinoma	Brain
968	+	Adenocarcinoma	Brain
969	+	Adenocarcinoma	Brain
970	+	Adenocarcinoma	Bone

NOS: not otherwise specified.

**Table 3:** RHAMM is upregulated in non-small cell lung carcinomas, especially in metastatic tumors with statistical significance

		RHAMM		
Tumor type	Total patients	Positive staining	Negative staining	*p* value
primary	156	89 (57%)	67 (43%)	< 0.001
metastatic	23	22 (96%)	1 (4%)	

### Expression pattern and prognostic value of *RHAMM* in lung adenocarcinoma of the TCGA cohort

**Figure 2:**
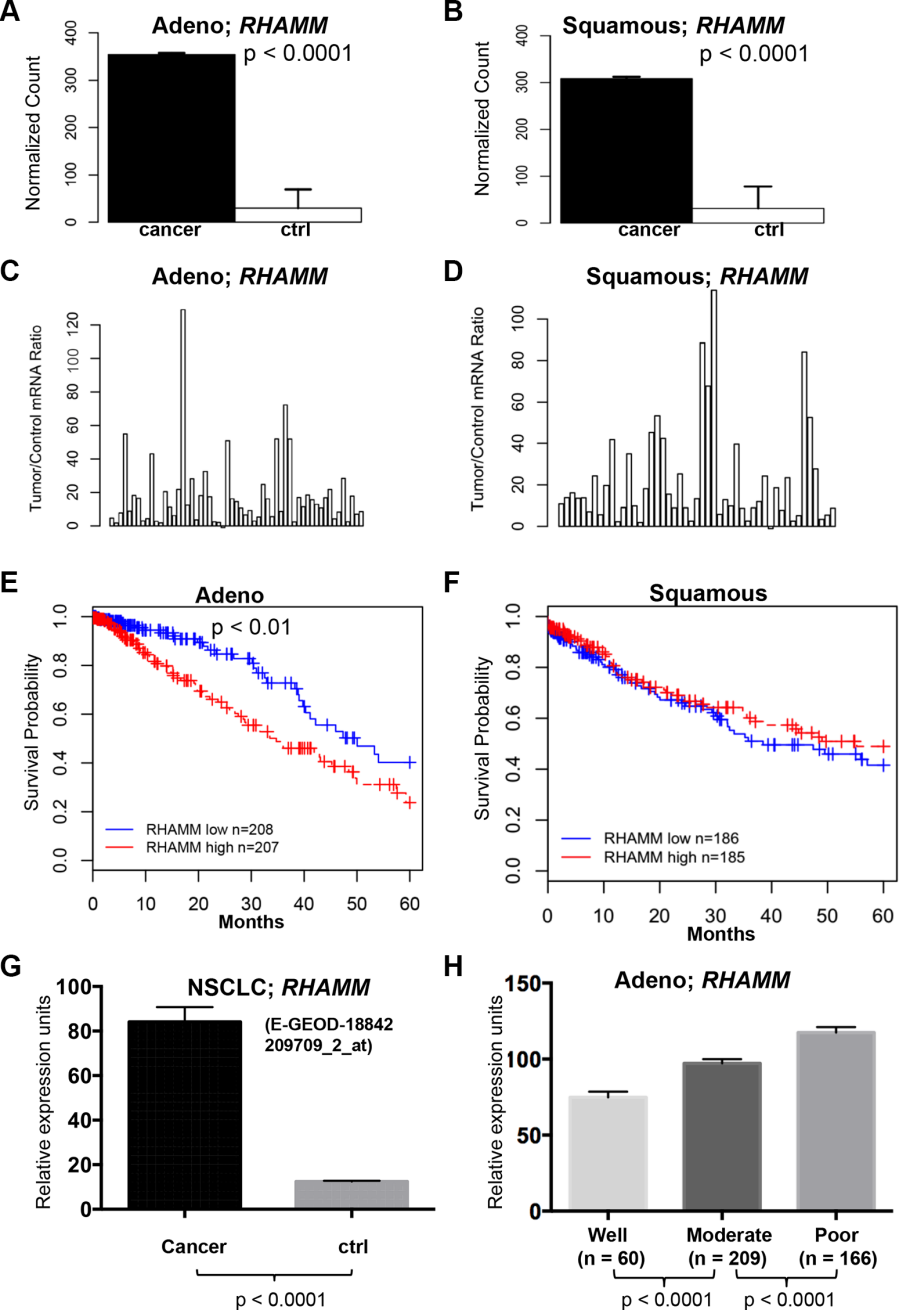
*RHAMM* gene expression is upregulated in lung adenocarcinomas compared to the patient-matched normal tissues and is associated with poor prognosis from TCGA datasets (**A–D**) The means and ratios of *RHAMM* RNA-Seq version 2 values from individual paired control to lung adenocarcinoma (A and C) and squamous cell carcinoma (B and D) of TCGA dataset are plotted. (**E**) Upregulation of *RHAMM* is associated with poor prognosis of the TCGA cohort with 415 lung adenocarcinomas. (**F**) Upregulation of *RHAMM* is not associated with poor prognosis of the TCGA cohort with 371 squamous cell carcinomas. High and low in the Figure legend represent the status of the *RHAMM* mRNA expression level compared to the median of the expression. (**G**) *RHAMM* is upregulated in NSCLC (ArrayExpress dataset E-GEOD-18842, [28]). The means and standard errors of *RHAMM* expression values from NSCLC (*n* = 46) and controls (*n* = 45) are plotted for comparison. The original log2 values from dataset are transformed into regular numeric values. Probe identification for *RHAMM* detection is 209709_s_at. (**H**) *RHAMM* gene expression in lung adenocarcinoma with various differentiated stages. Analysis of the Director's Challenge cohort dataset of 435 human lung adenocarcinoma cases that were well, moderately, or poorly differentiated. The graph represents the means and standard errors of cases in each differentiation category. The original log2 values from dataset are transformed into regular numeric values. Probe identification for *RHAMM* in Affymetrix microarray platform is 209709_s_at.

In order to determine whether *RHAMM* mRNA expression levels in NSCLC are also upregulated, we queried The Cancer Genome Atlas (TCGA), a publicly available database, for lung adenocarcinoma and lung squamous cell carcinoma datasets. In 57 paired lung adenocarcinomas and 49 paired lung squamous cell carcinomas, *RHAMM* gene expression (RNA-Seq version 2) was 12- and 10-fold higher in tumors than in matched normal tissues, respectively (*p* < 0.0001, Figure 2A and 2B). Fifty-three of 57 lung adenocarcinomas (94%) and 48 of 49 lung squamous cell lung carcinomas (98%) had at least 2-fold increased mRNA levels over the adjacent controls (Figure 2C and 2D). Survival analysis based on mRNA expression levels showed that higher *RHAMM* expression predicts poor outcomes in lung adenocarcinoma (*p* < 0.01, Figure 2E), but not in lung squamous cell carcinoma (Figure 2F). Upregulation of *RHAMM* in NSCLC was confirmed in the independent dataset from ArrayExpress E-GEOD-18842 [28] (Figure 2G). To further prove the relevance of *RHAMM* expression in NSCLC to clinical pathological stages, we used the Director's Challenge cohort with lung adenocarcinomas from caArray of National Cancer Institute for validation [29]. Consistent with the results from our IHC cohort, *RHAMM* mRNA expression in the Director's Challenge cohort correlates with tumor differentiation stage of lung adenocarcinomas (Figure 2H).

### *RHAMMv3 (RHAMM^B^)* expression in lung adenocarcinoma

**Figure 3:**
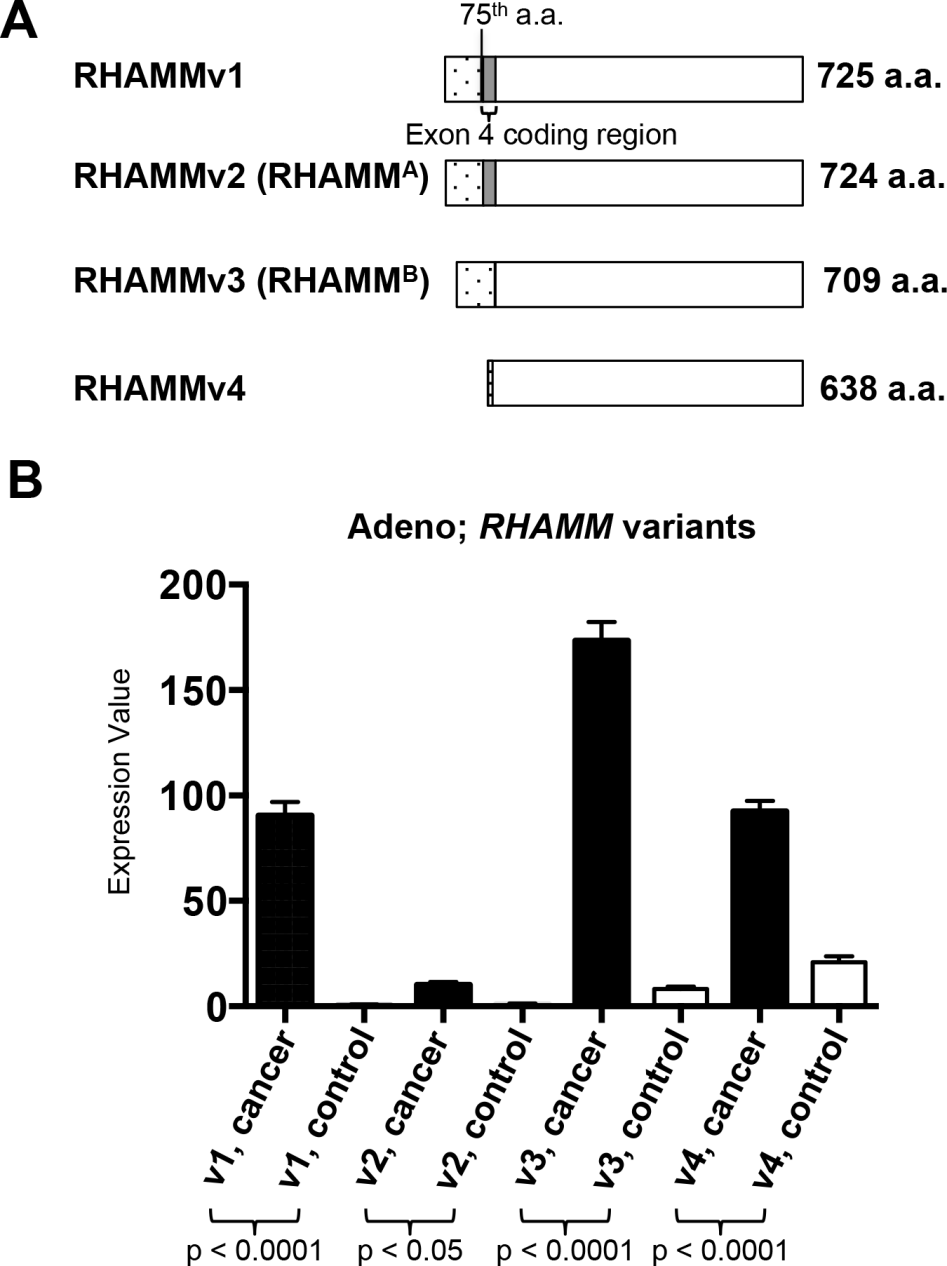
*RHAMM* variant gene expression from TCGA lung adenocarcinomas and normal lung tissues (**A**) Schematics of the 4 RHAMM variants. (**B**) The means and standard errors of 4 variants of *RHAMM* expression values from TCGA lung adenocarcinomas and normal lung tissues are plotted for comparison. Variant 1: uc003lzh or NM_001142556. Variant 2 (*RHAMM*^A^): uc003lzf or NM_012484. Variant 3 (*RHAMM^B^*): uc003lzg, or NM_012485. Variant 4: uc011dem or NM_001142557. Cancer, *n* = 470. Normal, *n* = 58.

The *RHAMM* gene encodes 18 exons and 4 unique variants are generated through alternative splicing (Figure 3A). *RHAMMv1* is the longest isoform encoding 725 amino acids ((NCBI RefSeq accession number: NM_001142556). *RHAMMv2* (NM_012484, also known as *RHAMM*^A^) uses an alternate acceptor splice site at one of the coding exons compared to transcript variant 1, resulting in an isoform that is 1 amino acid shorter than variant 1. *RHAMMv3* (NM_012485, also known as *RHAMM*^B^) lacks exon 4, a 45-nucleotide sequence [30].*RHAMMv4* (NM_001142557) misses 3 consecutive internal coding exons compared to transcript variant 1, which results in translation initiation from an alternate AUG start site and a shorter isoform with an unique N terminus compared to the other 3 variants.

We analyzed the transcript levels of the 4 *RHAMM* variants in the 58 normal lung tissues and 470 lung adenocarcinomas from the cohorts in the TCGA dataset. RNA-Seq data allows direct comparison of isoform abundance within one sample. Transcript levels of all 4 *RHAMM* variants were significantly higher in tumors compared to normal tissues, and *RHAMMv3* (*RHAMM*^B^) was the most prominent transcript (Figure 3B). Taken together, these results suggest that *RHAMM* is a prognostic factor for NSCLC especially for lung adenocarcinoma and *RHAMMv3* (*RHAMM*^B^) is the dominant variant in lung adenocarcinoma.

**Figure 4:**
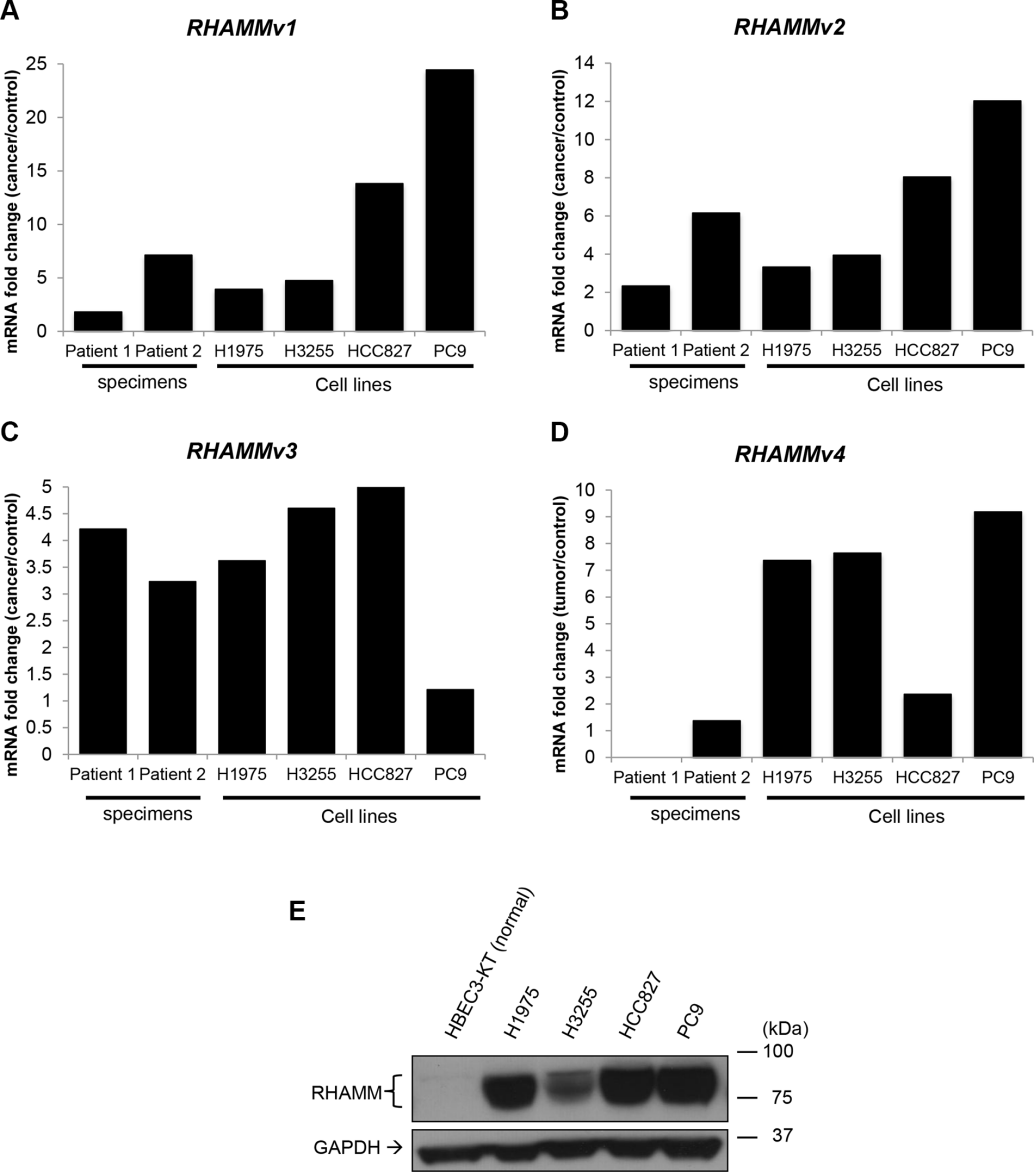
*RHAMM variant* mRNA and protein expression in human lung adenocarcinoma and cell lines (**A–D**) Transcripts of *RHAMM variant 1–4* detected by RT-qPCR. The ratios of mRNA expression value of tumor or cancer cell lines (H1975, H3255, HCC827, and PC9) to cancer adjacent controls or normal cell line are plotted. (**E**) RHAMM proteins detection by Western blot.

To independently validate the expression of *RHAMM* variants in lung adenocarcinoma, we performed real-time quantitative PCR using isoform specific primers for 2 lung adenocarcinoma and paired normal tissues, 4 lung adenocarcinoma cell lines (H1975, HCC827, H3255, and PC9), and a control lung bronchial epithelial cell line (HBEC3-KT). In agreement with TCGA results, we confirmed that the upregulation of *RHAMM* variants in tumors and cancer cell lines (Figure 4A–4D). We also observed the upregulation of RHAMM proteins in lung adenocarcinoma specimens and cancer cell lines in Western blot analysis using a pan-RHAMM antibody (Figure 4E). The predicted RHAMM sizes without post-translational modification are very close to each other (v1: 84.2 kDa, v2: 84.1 kDa, v3: 82.3 kDa, and v4: 74.5 kDa) and it is unknown whether any of RHAMM variants have post-translational modification in lung cells.

### Short hairpin RNA-mediated knockdown of *RHAMM* inhibits migration of human lung adenocarcinoma cell lines

**Figure 5:**
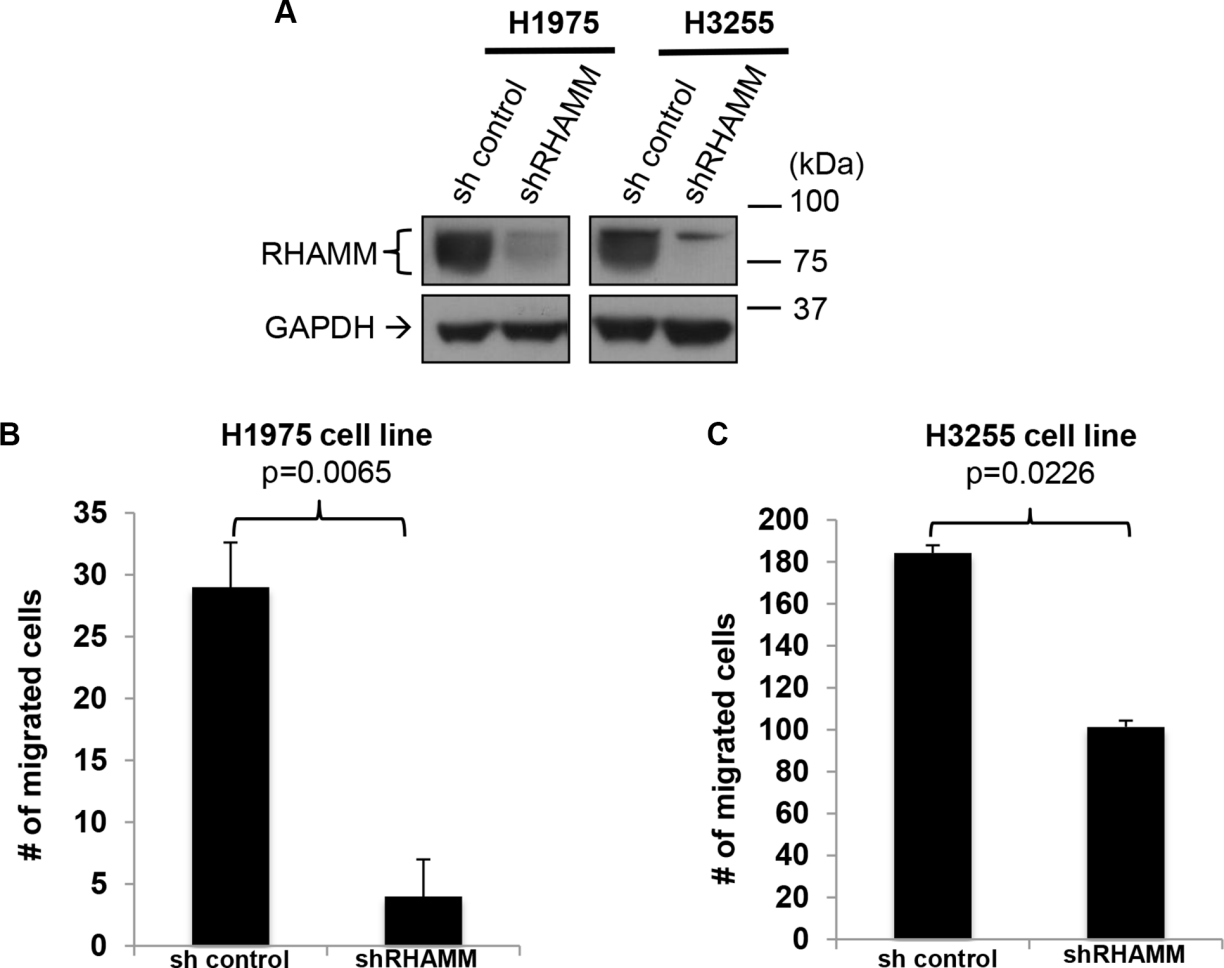
RHAMM is crucial for the migration capacity of human lung adenocarcinoma cell lines (**A**) Confirmation of RHAMM knockdown in H1975 and H3255 cell lines. Western blot analysis of RHAMM and GAPDH (as a loading control) in the two human lung adenoma cell lines. (**B** and **C**) Inhibition of H1975 and H3255 migration with sh*RHAMM* was determined using *in vitro* transwell migration chamber with a serum gradient (0% to 10%). Migrated cells on the bottom of the plate (B) or the bottom surface of the transwell inserts (C) were counted from 8 randomly picked fields under 10 × magnifications. Error bars represent s.e.m.

To assess whether RHAMM promotes migration of human lung adenocarcinoma cells, we performed a stable knockdown of *RHAMM* by short hairpin RNA (shRNA) in H1975 and H3255 cells. Since this shRNA targets a common sequence of RHAMM variants, expression of all *RHAMM* variants was expected to be reduced. Down-regulation of RHAMM was confirmed by Western blot analysis using a pan-RHAMM antibody (Figure 5A). We found that knockdown of RHAMM significantly inhibited the migration ability of human lung adenocarcinoma cell lines, H1975 (Figure 5B) and H3255 (Figure 5C).

## DISCUSSION

Despite improvements in therapy, lung cancer remains the leading cause of cancer-related mortality. NSCLC accounts for more than 80% of all lung cancer cases and the majority are metastatic upon diagnosis. The TNM staging is currently used for predicting the prognosis and treatment of patients with lung cancer. However, there is considerable variation in the prognosis of cancer in patients within the same stage [31]. The identification and characterization of the molecular changes during tumor progression and metastasis are critical for improving disease prevention, early detection, and treatment.

Recent studies have suggested that RHAMM is upregulated in cancer. Here, we report that while RHAMM protein expression is undetectable in normal lung tissue by immunohistochemistry, it is expressed in 57% of NSCLC (Figure 1 and Table 1). Consistent with the IHC data, the mRNA expression levels of *RHAMM* are significantly higher in lung adenocarcinoma and squamous cell carcinoma, compared to the patient-matched normal lung tissue (Figure 2A–2D). Moreover, this is the first study, to the best of our knowledge, which demonstrates the presence of RHAMM in the majority of distant NSCLC metastases and suggests a role for RHAMM in developing lung metastases (Figure 1 and Tables 2 and 3). We further demonstrate that shRNA knockdown of RHAMM inhibits tumor cell migration in two lung adenocarcinoma cell lines (Figure 5B and 5C).

We also show that RHAMM expression correlates with locally advanced primary tumors and poor differentiation in a TMA cohort of 156 patients (Table 1). Consistent with the IHC data from the TMA, we found that *RHAMM* mRNA expression correlated with less differentiation of lung adenocarcinomas in the Director's Challenge cohort dataset of 435 human lung adenocarcinoma cases (Figure 2H). There is no statistical difference in RHAMM expression with patients' age, sex, smoking history, or histological type of NSCLC in our TMA dataset (Table 1). In this TMA study, RHAMM protein expression was compared in patients with node-positive and negative primary NSCLCs. Although there was an increasing trend of RHAMM positivity in primary tumors with regional nodal metastases, it did not reach statistical significance and beckons study in larger cohorts of patients.

The survival analysis suggests that *RHAMM* mRNA is a prognostic factor for lung adenocarcinoma, but not for lung squamous cell carcinoma (Figure 2E and 2F). Similarly, activating mutations in EGFR are common in lung adenocarcinomas [32–34], but they are typically not present in squamous cell carcinomas [35, 36]. We have previously demonstrated that upregulation of RHAMM^B^ activates EGFR signaling [27]. It is possible that aberrant RHAMM^B^-EGFR signaling affects the survival of lung adenocarcinoma patients, but not that of squamous cell carcinoma patients. In addition, a recent study has reported that RHAMM protein expression is a negative prognostic factor in large cell lung carcinoma [37], which accounts for 10% of NSCLC.

The mRNA levels of all 4 *RHAMM* variants are upregulated in the lung adenocarcinomas compared to normal lung, with *RHAMMv3* (*RHAMM*^B^) being the predominant isoform (Figure 3B). Several studies have suggested the importance of *RHAMMv3* (*RHAMM*^B^) in tumorigenesis. First, a high ratio of *RHAMMv3* (*RHAMM*^B^)/*RHAMMv2* (*RHAMM*^A^) is a prognostic factor for multiple myeloma [38]. Second, an increasing trend between *RHAMMv3* (*RHAMM*^B^) transcript expression and advancing stage has been reported in untreated human B-cell chronic lymphocytic leukemia (B-CLL) patients [39]. Third, we demonstrated that RHAMMv3 (RHAMM^B^) significantly promotes liver metastasis in mouse models of pancreatic neuroendocrine tumors [27]. Here, we showed that knockdown of RHAMM inhibited the migration ability of human lung adenocarcinoma cell lines, H1975 and H3255. Taken together, expression of RHAMM is a valuable prognostic factor of lung adenocarcinoma, and our findings suggest that RHAMM, most likely RHAMM^B^, is a potential therapeutic target for preventing metastatic NSCLC.

## MATERIALS AND METHODS

### Clinical and pathological data

The study was approved by the New York Presbyterian Hospital/Weill Cornell Medicine Institutional Review Board. A tissue microarray (TMA) was constructed from a cohort of 156 patients with NSCLC resected at New York-Presbyterian Hospital/Weill Cornell Medicine between 1992 and 2007. This microarray was constructed using formalin-fixed, paraffin embedded tissue cores of 0.6 mm diameter (3 per tumor). The tissue cylinders were punched from morphologically representative tumor areas after review by a pathologist and brought into recipient blocks in triplicate using a semiautomated tissue arrayer. Non-neoplastic lung, benign lymph nodes and benign thymic tissue were incorporated into each microarray as controls. Twenty-three patients with metastatic NSCLC were identified in the database from 2009 to 2014. Patient demographics and clinical and pathologic staging (AJCC 7^th^ edition) were recorded.

### Immunohistochemistry and scoring of protein expression

Immunohistochemical staining (IHC) was performed using a RHAMM antibody [EPR4055] (Abcam, Cambridge, MA. Catalogue number: ab108339) or a Ki67 antibody (Dako, Carpinteria, CA. clone: MIB-1, Catalogue number: M7240) on paraffin embedded tissue sections on a Leica Bond system (Buffalo Grove, IL.) using the standard protocol F provided by the manufacturer. The section was pre-treated using heat mediated antigen retrieval with Tris-EDTA buffer (pH = 9, epitope retrieval solution 2) for 20 min and incubated with RHAMM antibody (1:100 dilution) or Ki67 antibody (1:50 dilution) for 15 min at room temperature. RHAMM or Ki67 were detected using an HRP conjugated compact polymer system and DAB as the chromogen. Each section was counterstained with haematoxylin and mounted with Leica Micromount. RHAMM expression for each carcinoma was given a score of 0 if no staining was present and a score of 1 if any cytoplasmic staining was present. For analysis of Ki67 positive cells, images were taken at 20× at a focus with the highest number of positive cells for each case, and analysis was done with Immunoratio [40].

### Bioinformatics and statistical analysis

Database and hospital records associated with the TMA of 156 patients were reviewed for demographic, clinical, and pathologic information. Descriptive statistics (including frequency, percent, median, interquartile range) are presented for demographic and pathological characteristics. Variables of interest were examined by the *χ*^2^ test for categorical variables and Mann-Whitney *U* test for continuous variables. All analyses were performed in IBM SPSS statistics 22 (SPSS Inc., Chicago, IL).

Bioinformatics and other statistical analyses were conducted on the publicly available gene expression datasets (accessed July 2014) from The Cancer Genome Atlas (TCGA; http://cancergenome.nih.gov/), lung adenocarcinoma microarray data from caArray (https://array.nci.nih.gov/caarray/home.action) [29] and NSCLC microarray data from ArrayExpress (https://www.ebi.ac.uk/arrayexpress). TCGA datasets with RNA-Seq version 2 gene expression values (level 3) and clinical data with patients' survival information from lung adenocarcinoma and lung squamous cell carcinoma, caArray and ArrayExpress datasets (Affymetrix GeneChip Human Genome U133 Plus 2.0 format) with expression values were directly downloaded from the above websites and analyzed using statistical programing software R (version 2.14.1) or statistical software Prism (version 6.0f). Two-tailed Mann-Whitney *U* test was used to compare differences between two groups selected. *P* value < 0.05 is considered as statistical significance. Gene expression comparison (RNA-Seq version 2) is performed using normalized counts (cancer and adjacent normal) or expression values.

### Quantitative real-time reverse transcription PCR (RT-qPCR)

Frozen human tumors and adjacent non-neoplastic lung samples were obtained from the Cardiothoracic Surgery Department, Weill Cornell Medicine. Specimens were collected after obtaining written informed consent prior to undergoing any study-specific procedures in accordance with the Declaration of Helsinki. Patient's identity of pathological specimens remained anonymous in the context of this study. Patient sample collection was approved by the Institutional Review Board of Weill Cornell Medicine.

mRNA was isolated from frozen human specimens or cell lines grown on 6-cm or 10-cm plates using RNeasy mini kit (Qiagen) containing gDNA Eliminator spin columns. The cDNA from frozen human specimens was generated using SuperScript III First-strand synthesis system with random hexamers (Invitrogen) and cDNA from cell lines was generated using SuperScript ViLo kit with random hexamers (Invitrogen). Power SYBR green (Invitrogen)-based RT-qPCR was performed with 2 internal control genes and the comparative C_T_ method (ΔΔC_T_) (ABI).

The sequences of the primers used are *RHAMMv1* (forward: 5′- AGATACTACCTTGCCTGCTTCAG-3′, reverse: 5′-CTTTATCATTCTTTTGAGATTCCTTC-3′)_;_ *RHAMMv2* (forward: 5′- tgacaaagatactacct tgcctgct-3′, reverse: 5′-tcattcttttgagattcctttgattc-3′); *RHAMMv3* (forward: 5′-AAAGTTAAGTCTTCG GAATCAAAGATT-3′, reverse: 5′- GCATTATTTGCA GAGAGAGATGT-3′); *RHAMMv4* (forward: 5′- ATGACCCTTCTGATTCGTGTTC-3′, reverse: 5′- GCCTTGCTTCCATCTTTTCCA-3′); and internal control genes: human *HMBS* (forward: 5′-CCATCATCCT GGCAAC AGCT-3′, reverse: 5′-GCATTCCTCAGGGTGCAGG-3′); and human *MRPL19* (forward: 5′-GGGATTTGCATTCAG AGATCAGG-3′, reverse: 5′-CTCCTGGACCCGAGGAT TATAA-3′).

### Cell lysates and Western blotting

Whole-cell lysates were prepared using RIPA buffer containing 1 mM dithiothreitol, 1 mM sodium orthovanadate, 10 μL/mL of EDTA-free, protease inhibitor cocktail (Sigma), 1 mM PMSF and 100 nM okadaic acid. After addition of lysis buffer cocktail, samples were rotated at 4°C for 30 minutes and subsequently passed through a syringe with a 27G needle five times. Samples were then centrifuged for five minutes at 13,000 rpm in a microcentrifuge to remove debris and supernatant collected. Protein was quantitated using a Bradford assay (BioRad). Absorbance was measured using a BioRad SmartSpec 3000.

For Western blot analysis, 60 μg of cell extracts were loaded into Nu-PAGE Novex 4–12% Bis-Tris pre-cast gels (Life Technologies). Protein was immobilized onto PVDF membrane, 0.45 μm pore size (Life Technologies). Blots were blocked for one hour with 3% (weight/volume) bovine serum albumin (Fisher Scientific) and incubated overnight at 4°C with a rabbit monoclonal antibody to RHAMM [EPR4055] antibody at 1:1,000 dilution (Abcam, Cambridge, MA. Catalogue number: ab108339). The next day blots were washed 4 times for 10 minutes with TBST and incubated for one hour at room temperature with an anti-rabbit secondary antibody at 1:5,000 dilution (R&D Systems). Bands were detected with enhanced chemiluminescence (Fisher Scientific).

### shRNA knockdown of RHAMM

Lentiviruses harboring shRNA targeting *RHAMM* (Sigma, Clone ID: NM_012484.2-2128s21c1, GCCAACTCA AATCGGAAGTAT) or a scrambled, non-targeting sequence (Sigma, shc216) were generated using 293T cells as described previously [41]. Cells were seeded at a density of 250,000 (H1975) or 500,000 (H3255) cells/well in 6-well plates and infected with lentivirus after 24 hours at a MOI of 5, using 10 μg/mL polybrene. Antibiotic selection was initiated 48 hours after infection with 5 μg/mL puromycin in RPMI1640 medium and later reduced to 1 μg/mL.

### *In vitro* transwell cell migration assay

The Transwell cell migration assay was modified based on previously described protocols [42, 43]. Briefly, 2 × 10^5^ of H1975 cells with shRNA (for non-target control or RHAMM) or 1 × 10^5^ of H3255 cells with shRNA (for non-target control or RHAMM) were seeded in the upper chambers of 8- porous polycarbonate membranes (Corning, 3422) with serum-free RPMI1640. The lower chambers were filled with RPMI1640 containing 10% FBS, 0.2 mM L-glutamine, and 1% penicillin/streptomycin. After 16 h incubation for H1975, cells migrating to the bottom of the wells were fixed with 4% PFA, stained with 0.1% crystal violet for 30 min, destained, visualized microscopically, and photographed. After 17.5 h incubation for H3255, cells migrating to the bottom surface of the transwell inserts were fixed with 4% PFA, stained with 0.1% crystal violet for 30 min, destained, visualized microscopically, and photographed. Migrated cells in each transwell were counted in 8 fields under 10× magnification.

## Abbreviations

HA: Hyaluronic acid
RHAMM: Receptor for hyaluronic acid-mediated motility
NSCLC: Non-small Cell Lung Carcinoma
Adeno: lung adenocarcinoma
Squamous: Squamous cell carcinoma
IQR: interquartile range
Nx: patients in whom the regional lymph nodes cannot be assessed
No: no regional metastases detected
N+: regional lymph node metastases
NOS: not otherwise specified
met: metastasis
TMA: tissue microarray
RT-qPCR: Quantitative real-time reverse transcription PCR
shRNA: short hairpin RNA
